# Surgical resection of pulmonary metastases from colorectal cancer: 11 years of experiences

**DOI:** 10.1371/journal.pone.0175284

**Published:** 2017-04-10

**Authors:** Guoquan Cao, Dezhi Cheng, Lechi Ye, Yiyuan Pan, Fan Yang, Shixu Lyu

**Affiliations:** 1 Department of Radiology, The First Affiliated Hospital of Wenzhou Medical University, Wenzhou, China; 2 Department of Cardiothoracic Surgery, The First Affiliated Hospital of Wenzhou Medical University, Wenzhou, China; 3 Department of surgical oncology, The First Affiliated Hospital of Wenzhou Medical University, Wenzhou, China; Institut national de la recherche scientifique, CANADA

## Abstract

**Objective:**

To analyze the benefits and prognostic factors after surgical resection of pulmonary metastases from colorectal cancer (CRC).

**Methods:**

From Jan. 2004 to Jan. 2015, continuous 88 cases diagnosed with pulmonary metastases from CRC, including 15 cases of synchronous metastases and 73 metachronous metastases, were analyzed in the retrospective study.

**Results:**

All of these 88 cases underwent curative pulmonary resection including 8 cases of simultaneous surgery. The one-year, three-year and five-year survival of the 88 cases were 93.4%, 60.2% and 35.7%, respectively. 63 patients just have one metastasis, and 25 patients have more than one metastasis. Additionally, the one-year, three-year and five-year survival was 98.1%, 70.2% and 40.3% respectively in one metastasis group, while 80.1%, 37.9% and 22.5% respectively in more than one metastasis group (p = 0.003). DFS of 37 metachronous metastases were equal or greater than 18 months, and DFS of 36 metachronous metastases were less than 18 months. The one-year, three-year and five-year survival was 97.8%, 77.9% and 41.4% respectively in the DFS≥18 month group, while 88.2%, 44.6% and 28.1% respectively in the DFS<18 month group (p = 0.01).

**Conclusion:**

Surgical resection of pulmonary metastases from colorectal cancer can improve survival rate in selected patients. It seems that the number of metastases is an independence prognostic factor in surgical treatment. Furthermore, longer DFI implies longer survival for resectable CRC pulmonary metastases.

## Introduction

Distant metastases, especially liver and lung, are nearly the most significant prognostic factor for colorectal cancer (CRC). Lung is the secondary frequent target organ for metastasis, and meanwhile, pulmonary and hepatic metastases are regarded as the limited diseases in some studies [1]. Though chemotherapy, targeted therapy, radiofrequency ablation and other new progressed therapeutic methods are applied in the treatment of pulmonary metastases from CRC, the five-year overall survival rate is still no more than 5% by systemic therapy[2]. Surgical resection is widely accepted as the choice for tackling pulmonary metastases from CRC[3, 4]. However, there are still arguments on surgical indications and prognostic factors[5–8]. In recent years, the indication for pulmonary resection, such as resection of recurrent lung metastases[9], has become broad.

This retrospective study analyzed continuous 88 patients of pulmonary metastases from CRC who accepted pulmonary resection. The objective of the study is not only to summarize our experiences with pulmonary resection of metastases tumors from colorectal cancer but also to evaluate prognostic factors which may guide the choice of surgical indications.

## Material and methods

### Patients

A retrospective analysis was performed to identify all pulmonary metastases patients with colorectal cancer who underwent pulmonary resection between Jan. 2004 and Jan. 2015. The clinical and follow-up data of patients were obtained from the hospitals’ medical records. A total of 88 patients were involved in this study, and the detailed clinical data can be summarized in Table 1. The study was approved by clinical research ethics board of Wenzhou Medical University. (NO. 2016–197).

**Table 1:** Clinical data.

Variable	n
Age(y,mean and range)	61.25(43–82)
Sex	
Male	50
Female	38
CEA level	
CEA≥5ng/ml	37
CEA<5ng/ml	51
DFS	
≥18	37
<18	36
metastases	
No. = 1	63
No.>1	25
synchronous metastasis	15
metachronous metastasis	73
Primary tumor	
rectum	46
colon	42

### Treatment strategy

Every patient underwent pulmonary resection as well as other treatments such as chemotherapy and targeted therapy on a case-by-case basis. Simultaneous excision was applied in 8 patients. Furthermore, staged excision was applied in 80 patients, including 7 synchronous metastasis patients and 73 metachronous metastasis patients. 25 cases had more than one metastases. 21 cases had monolateral lesions and 4 cases had bilateral lesions. In the 4 bilateral cases, 1 case operate in sequential bilateral procedure. The interval time between the two lung resections of another three cases was 28 days, 36 days and 42 days respectively. Before the secondary operation, examination were applied to ensure there were no other metastases appeared during the interval. The disease free survival time was calculated from the second operation.All of the patients applied R0 resection of pulmonary metastases. Moreover, all of the 88 patients followed the same surgical indication: if the metastases are resectable and only restricted in lung, the primary tumor and the metastases tumor can be R0 resected. Sufficient respiratory compensation should be given after resection. The treatment data can be summarized in Table 2.

**Table 2:** Treatment data.

Variable	n
Surgery	88
wedge resection	64
segment resection*	24
Simultaneous excision	8
Staged excision	80
Thoracotomy	20
VATS	68
chemotherapy	79
Oxaliplatin+5-FU	17
irinotecan+5-FU	35
Oxaliplatin+irinotecan+5-FU	12
5-FU	15
Targeted therapy**	21
cetuximab	12
bevacizumab	9

*: Including lobectomy

**: All targeted therapy applied with chemotherapy.

5-FU including fluorouracil and capecitabine

VATS: Video-assisted Thoracoscopic Surgery

### Statistical analysis

Survival time was calculated from the first pulmonary resection to death or the last contact during follow-up. DFS in metachronous metastasis patients means disease free survival between operation of primary tumor and appearence of pulmonary metastasis. Beyond that, median and actuarial survival was calculated using the Kaplan-Meier method. Furthermore, the relationships of possible prognostic factors to outcomes were calculated utilizing the log-rank method. It was considered being statistically significant when the p value is no more than 0.05. Moreover, the analysis was performed using the software of SPSS 16.0. The related survival curves could be drawn up by GraphPad Prism 5.

## Result

### Survival and prognostic factors

The median survival of 88 patients was 38 months, and one-year, three-year and five-year survival rate was 93.4%, 60.2% and 35.7%, respectively. Moreover, the median survival of 73 metachronous metastasis patients was 41 months, and one-year, three-year and five-year survival rate was 92.8%, 59.1% and 37.0% respectively. Median survival of 15 synchronous metastasis patients was 34 months, and one-year, three-year and five-year survival rate was 93.4%, 66.8% and 25.8%, respectively.

**Fig 1:**
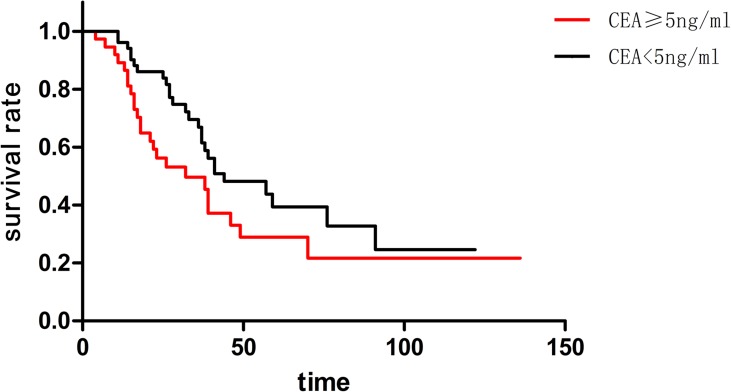
Though it seemed the CEA<5ng/ml group had a better behavior in survival than CEAastases. Mected patients. I"5 months, and 36 months or moreated.

The CEA level of 51 patients was lower than 5ng/ml, and the CEA level of 37 patients was equal or higher than 5ng/ml. The median survival of the two groups was 44 months and 32 months, respectively. Furthermore, the one-year, three-year and five-year survival rate of CEA<5ng/ml group was 96.7%, 69.1% and 42.5%, respectively. Beyond that, one-year, three-year and five-year survival rate of CEA≥5ng/ml group was 89.1%, 49.2% and 28.2%, respectively. Though the CEA<5ng/ml group apparently had a better behavior in survival, there was no statistical significance (p = 0.068) (Fig 1).

**Fig 2:**
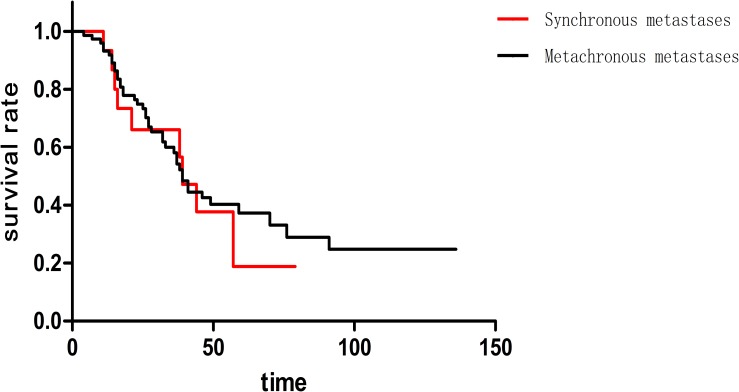
There was no statistical significance (p = 0.676) between synchronous metastasis patients metachronous metastasis patients.

**Fig 3:**
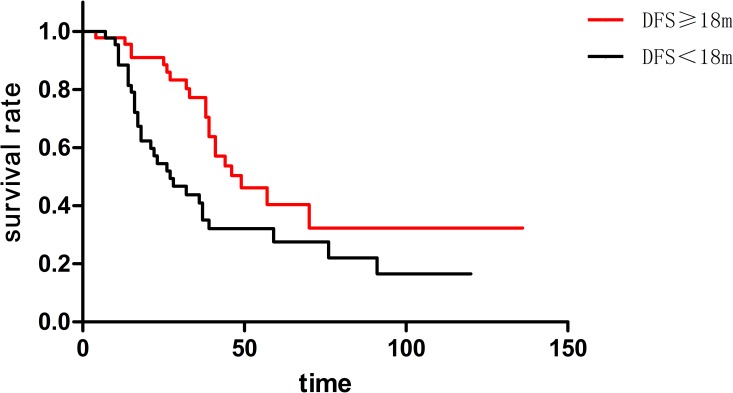
Longer DFS means longer survival in metachronous pulmonary metastases patients (p = 0.011).

This study involved 15 synchronous metastasis patients and 73 metachronous metastasis patients. The median survival of two groups was both 39 months coincidently. In addition, one-year, three-year and five-year survival rate was 93.4%, 66.8% and 25.8% respectively in synchronous metastasis patients, while 92.8%, 59.1% and 37.0% respectively in metachronous metastasis patients. There was no statistical significance (p = 0.676) (Fig 2) in these two groups. The median disease free survival (DFS) was 18 months among 73 cases of metachronous metastases patients. Moreover, the 73 patients could be divided into two groups, including 37 cases of DFS≥18 month and 36 cases of DFS<18 month. Here, DFS (disease free survival) means the interval between the primary tumor operation and the appearance of pulmonary metastases. Median survival of DFS≥18 months and DFS<18 months cases was 49 months and 27 months, respectively. Additionally, one-year survival rate was 97.8% and 88.2% respectively; three-year survival rate was 77.9% and 44.6% respectively; five-year survival rate was 41.4% and 28.1% respectively (p = 0.011)(Fig 3). The longer DFS means longer survival in metachronous pulmonary metastases patients.

**Fig 4:**
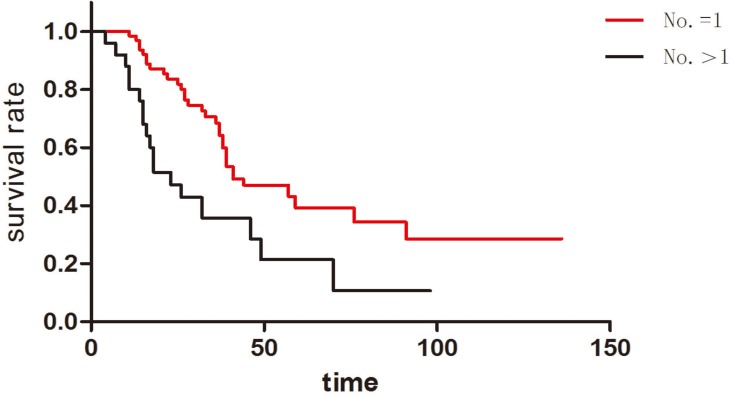
Patients with isolated pulmonary metastasis had longer survival than those whose metastases number were more than one (p = 0.003).

In this study, 63 patients had isolated pulmonary metastasis and 25 patients had more than one pulmonary metastases. The median survival of these two groups was 41 months and 23 months respectively. Furthermore, the one-year, three-year and five-year survival rate of metastases number = 1 group was 98.1%, 70.2% and 40.3% respectively. The one-year, three-year and five-year survival rate of metastases number>1 group was 80.1%, 37.9% and 22.5% respectively. Notably, there was statistical significance between these two groups (p = 0.003) (Fig 4). Compared to those with more than one metastases number, the patients with isolated pulmonary metastasis had longer survival after the pulmonary resection.

### Postoperative mortality, morbidity and recurrence

There were three postoperative severe complications evaluated by Clavien-Dindo Grade[10]. Stomal leak occurred in 1 simultaneous excision patient, and pulmonary infarction happened in 1 patients. Moreover, 1 patient experienced a severe thoracic infection. No one died for the surgery causes, and other minor complications could be controlled clinically

The median time to recurrence after pulmonary resection was 15 months (range: 3–72 months). To be specific, 51 patients (58.0%) experienced recurrence and totally 77 recurrences occurred. Additionally, 16 patients experienced two different recurrences and 3 patients experienced more than two different recurrences. The majority of recurrent tumors were identified mostly in the lung and liver, while the other recurrent sites included bones, primary tumor site, lymph node, and brain. (Table 3).

**Table 3:** Postoperative recurrence.

Variable	Recurrence No.
Lung recurrence	36
Liver recurrence	34
Bone recurrence	2
Primary tumor site recurrence	2
Lymph node	2
brain	1
total	77

## Discussion

Colorectal cancer is a systematic disease and the surgery is not the only method for treatment from the very beginning. However, surgery is almost the most effective way for cure. It’s really hard to design a control study between surgery and other therapeutic methods, such as chemotherapy, targeted therapy or radiofrequency ablation. Meanwhile, the randomised trials are insufficient and the evidence for pulmonary resection is weak theoretically[11]. Most of researches focused on surgical intervention, and some pay attention to other treatments for patients without surgical indications[12–14]. Little studies on the comparison of surgery and systemic therapy draw the obvious conclusion that the surgery is superior[15]. Indeed, all of these therapeutic methods are not isolated individuals and they supplement each other. In this study, chemotherapy was applied in 79 patients, and targeted therapy was applied in 21 patients. Two patients applied radiofrequency ablation after surgery because of pulmonary metastases recurrence. Furthermore, one patient was diagnosed with supraclavicular lymph node metastasis 7 months after the pulmonary wedge resection. Following a 40 GY local radiotherapy, the metastatic lymph node disappeared in the CT scan and B ultrasound.

Thoracotomy surgery and VATS refer to two different means in pulmonary resection. Which is superior? The criticism of VATS mainly focused on inability of palpation during resection, omission of small metastases, and probably un-adequate margins. To date, most of reports suggest optimistic outcomes for VATS in survival rate compared to thoracotomy surgery[16–18]. Furthermore, Nakajima et al. retrospectively analyzed 143 patients (72 patients underwent thoracoscopy and 71 thoracotomy) for pulmonary metastases from CRC[18]. According to their finding, there was a higher five-year recurrence-free rate in thoracoscopy (34.4%) than in thoracotomy (21.1%). However, it was not significant in overall five-year survival rate (49.3% & 39.5%, respectively). In this study, 20 patients applied thoracotomy and 68 patients applied VATS. Surgical options did not influence the survival rate and recurrence. The advantage of VATS was to decrease the hospital stay and operation time. But thoracotomy could save the hospitalization cost. Two pulmonary related severe complications occurred in thoracotomy group. A stomal leak occurred in a simultaneous excision VATS patient. (Table 4)

**Table 4:** Difference between the thoracotomy group and the VATS group.

Variable	Thoracotomy (n = 20)	VATS (n = 68)
Metastases No. = 1	14	49
Metastases No.>1	6	19
Operation time(min)*	85±24	76±18
Hospital stay(day)**	11±2	7±4
Operation fee(¥)**	15477±2321	26132±1175
Severe complications	2	1***

*It was subject to the longer time in staged bilateral metastases operation and simultaneous excisions were not included.

**Patients with severe complications were not included.

***Stomal leak happened in a synchronously resection of rectal cancer and pulmonary metastasi

Serum CEA level was considered as an independence prognostic factor in surgical treatment for pulmonary metastases from CRC in some clinical reports [8, 17, 19, 20]. In this study, the mean survival and median survival in CEA<5ng/ml group were better than CEA≥5ng/ml group. Notably, there was no statistical significance in these two groups. However, we still believe that serum CEA level should be regarded as an important prognostic factor. CEA changed in different disease periods. As a common practice, the test result was used before pulmonary resection. Will the differentiation of CEA level among different disease periods make different senses? This was another issue that should be discussed.

DFS was another independence prognostic factor in most of reports[10, 21, 22]. Renaa divided patients who had pulmonary metastases from CRC into three groups according to the DFS of 0–11 months, 12–35 months, and 36 months or more. According to their finding, DFS≥36 months had better prognosis than the others, yet the difference was significant (p = 0.0317)[21]. Also, they were divided into three groups in accordance with group one DFS≥36 months and CEA<5ng/ml, group two DFS<36 months or CEA>5ng/ml, group three DFS<36 months and CEA>5ng/ml. They found that group one had significant better (p = 0.0001) prognosis than the other two groups. According to the finding of this study, the longer DFS implies longer survival in metachronous pulmonary metastases patients. Meanwhile, the conclusion indicates that the shorter DFS patients had no need to accept operation. Furthermore, we recommend pulmonary resection for those patients only if they are eligible.

Multiple pulmonary metastases were another poor prognostic factor in this study, and this factor was mentioned in many other similar studies[23].As a matter of fact, in some solitary metastases cases, some occult metastases may not be found before pulmonary resection by CT or PET-CT. However, these occult metastases could be eliminated by other therapeutic methods. For example, the chemotherapy around the surgery is easier than those distinct metastases. There was another special situation: some lesions were probably not real metastases tumors but inflammatory nidus, lymph nodes, or dysplastic nodules. Thus, the problem was not the number of metastases. Indeed, the problem was whether all the metastases could be completely removed by surgical procedure and other systemic or regional therapeutic methods.

Otherwise, there were several limitations in this study. Some data, such as tumor size, perioperative imaging and pathological materials, were imperfect. Thus, related data could not be analyzed in this study. Meanwhile, this study had a time span of more than 11 years. During the past 11 years, the medical technology, chemotherapy medicine and treatment idea changed a lot. As a result, not all the patients allowed the same therapeutic standards. This study might be influenced by these limitations. Thus, more clinical data are needed to get more significant analysis.

## Conclusion

The surgical resection of pulmonary metastases from colorectal cancer can improve survival rate in selected patients. It seems that the number of metastases is an independence prognostic factor in the surgical treatment. Longer DFS implies longer survival for resectable CRC pulmonary metastases. Moreover, serum CEA level should be considered as another potential prognostic factor.
